# Broadband Insulator-Based Dynamic Diode with Ultrafast Hot Carriers Process

**DOI:** 10.34133/2022/9878352

**Published:** 2022-09-13

**Authors:** Runjiang Shen, Yanghua Lu, Xutao Yu, Qi Ge, Huiming Zhong, Shisheng Lin

**Affiliations:** ^1^College of Information Science and Electronic Engineering, Zhejiang University, Hangzhou 310027, China; ^2^Chongqing 2D Material Institute, Chongqing 410020, China; ^3^Department of Emergency, The Second Affiliated Hospital, Zhejiang University School of Medicine, Zhejiang University, Hangzhou 310009, China; ^4^State Key Laboratory of Modern Optical Instrumentation, Zhejiang University, Hangzhou 310027, China; ^5^Hangzhou Gelanfeng Technology Co. Ltd., Hangzhou 310051, China

## Abstract

The excitation, rebound, and transport process of hot carriers (HCs) inside dynamic diode (DD) based on insulators has been rarely explored due to the original stereotyped in which it was thought that the insulators are nonconductive. However, the carrier dynamics of DD is totally different from the static diode, which may bring a subverting insight of insulators. Herein, we discovered insulators could be conductive under the framework of DD; the HC process inside the rebounding procedure caused by the disappearance and reestablishment of the built-in electric field at the interface of insulator/semiconductor heterostructure is the main generation mechanism. This type of DD can response fast up to 1 *μ*s to mechanical excitation with an output of ~10 V, showing a wide band frequency response under different input frequencies from 0 to 40 kHz. It can work under extreme environments; various applications like underwater communication network, self-powered sensor/detector in the sea environment, and life health monitoring can be achieved.

## 1. Introduction

Dynamic semiconductor physics has emerged as the platform of exploring the novel semiconductor devices since the dynamic Schottky diode has been proposed [1, 2]. Since the discovery of DD in 2018 [1], the novel and ultrafast mechanical-electrical coupling process at the solid-solid/solid-liquid interfacial barrier has been taken seriously due to groundbreaking physical picture, especially the unusual excitation, rebound, and transport of HCs induced by the mechanical input, which has brought the births of various evidences including the dynamic PN/Schottky/heterojunction [1, 3–10], polarized solution based DD [11, 12], tunneling DD [2, 10], and nonlinear synergy DD [13–16]; some excellent characters like direct-current output, high current density, flexibility, suitable inner resistance, and easy fabrication have also been proven [1, 2, 5], and the integrable ability has also been illustrated in recent works [17–19]. These devices open an exciting new avenue for recycling in situ mechanical energy efficiently, showing great potential to subvert the current in-situ energy harvest system as the macroscopic electricity output density of these generators has reached over 100 W/m^2^ [3, 17, 20–22]. Besides, the ability to work under extreme conditions is also systematically illustrated [23]. However, more experiments should be illustrated to push forward and optimize the underlying physics about the DD, especially the carrier process inside traditional insulators, and the output voltage needs further enhancement. The physics inside DD is counterintuitive and different from the static diode, Thus, DD is a perfect platform to explore the process of HCs inside insulators, while the static semiconductor physics usually treat an insulator as a nonconductive material as a consequence of the extremely large bandgap. The transport of electrons inside insulators is normally inhabited by the strong scattering process. In the framework of DD, the rebounding process can induce HCs otherwise diffused electrons, which promises the really high energy of those electrons and may even permit the crossover of these HCs through the insulators; however, no such experiment has been carried out.

Herein, systematic research of the HC process inside insulators is illustrated here by applying the vertical DD based on a semiconductor/insulator, which breaks the traditional solid boundaries between insulators/conductors defined by the conductivity. With the excitation, rebound at the semiconductor/insulator interface, and transport inside insulators/semiconductors, HCs can remain at a high energy level before reaching electrodes, leading to a higher voltage output. A DD device based on a silicon (Si)/insulator (fluorinated ethylene propylene (FEP)) structure shows an ultrafast response time of ~1 *μ*s and voltage output of ~10 V under the input frequency of 40 kHz. More inspiringly, the mechanical input mode can affect the response time, and a wide spectrum is also illustrated. The device performance shows a high consistency between a wide band range from 0 to 40 kHz with the stable voltage output of ~10 V, and it can covert mechanical input signal into electrical signal in the same frequency, which is firstly reported in the related areas. The excellent performance is better than previous self-powered sensors in most previous reports, and we predict the fastest response time can be less than 1 ns with the input frequency in the order of GHz/THz. This work opens up an exciting avenue of utilizing the potential for combining mechanical signal to the self-powered sensor network and electronic communication system under various extreme conditions, including ocean, land, and air.

## 2. Results and Discussion

**Figure 1:**
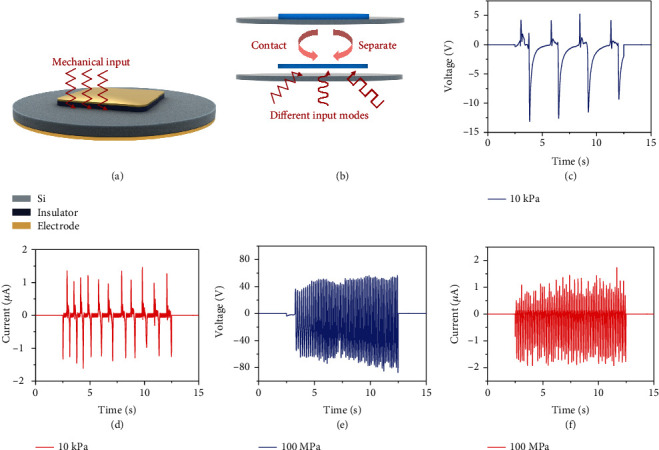
Basic structure and generation process of DD based on semiconductor/insulator structure. (a) 3D schematic diagram of the device at rest, the structure selected here is metal (also as top electrode)/insulator/semiconductor/back electrode; the mechanical signal inputs into the interface of insulator/semiconductor. (b) Schematic diagram of a circle of electrical generation process with different input signal modes with the contact-separation movement. (c) Typical voltage output under the pressure of 10 kPa. (d) Typical current output under the pressure of 10 kPa. The pressure here is provided by normal human touch, which is close to triangle signal mode. (e) Typical voltage output under the pressure of 100 MPa. (f) Typical current output under the pressure of 100 MPa. The pressure here is provided by extreme human touch, which is close to step signal mode.

Experimental results are detailed illustrated below.  Figure 1(a) shows the 3D schematic diagram of the static device structure. The order of the materials from the bottom to the top is gold (back electrode of semiconductor), semiconductor (Si), insulator, and metal (also as top electrode). The mechanical input is introduced to the device at the interface between semiconductor/insulator in the form of different waves with high entropy. A cycle of electrical generation consists of contact-separate (or press and release) as illustrated in Figure 1(b). Different modes of mechanical wave play the role as mechanical signal input, and the electrical signal output occurs with the generation and rebound of mechanically excited HCs. A notable phenomenon is the different relations of voltage/current and mechanical pressure input. While the input pressure increases, voltage output shows a highly correlated relations; however, current does not improve significantly. Under the input conditions allowed by the experiment, the maximum electricity output for FEP/p-Si-based DD is reached at 100 MPa pressure. Voltage amplitude of ~50 V and current of ~1 *μ*A for a single device is observed. We suppose this can attribute to the limited amounts of HCs inside insulators, which may hinder the transport part of the generation process. However, the higher input energy can be fully utilized to excite HCs, making the HCs to a higher level in the excitation part, which equals a higher energy level can be reached for a single rebounded carrier; thus, they maintain higher energy after transport to output higher voltage. But the carrier concentration of insulators is a limited constant, and experimental data reveals that nearly all movable carriers have been rebounded, leading to a constant amount of HCs, which is equal to the current generation. Unlike previous DD based merely on semiconductors/metals/thin tunneling layer (<1 *μ*m), DD based on thicker insulators (>10 *μ*m) shows a higher voltage output, and the frequency response characteristics are fully investigated and illustrated, Interestingly, even the commonly used insulation tape can also make up the device as shown in Figure S7. We suppose this difference originates from the special rebound and hopping process of mechanically excited hot carriers through the defects of insulators, and the contact-separation input mode leads to the alternative current output.

**Figure 2:**
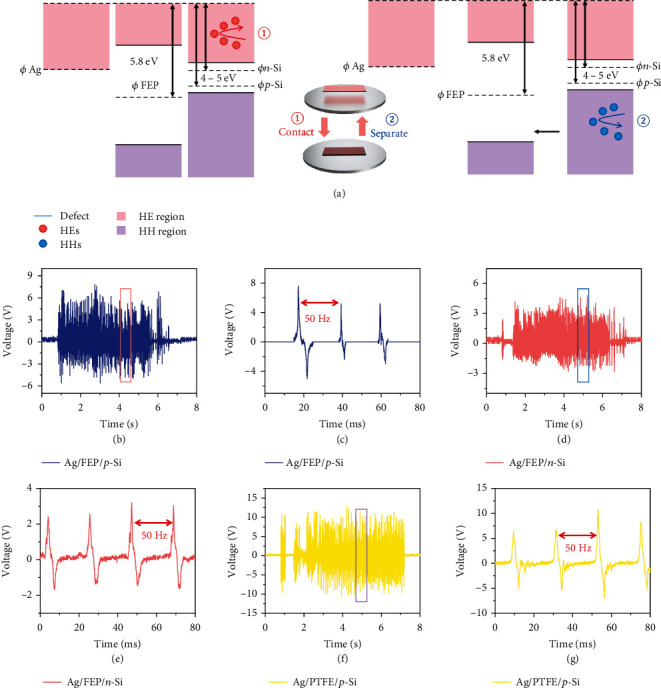
Schematic illustration of the mechanism of DD based on insulator. (a) Proposed energy band diagram and carriers transport inside DD based on insulators/semiconductors heterostructure with the (1) contact-(2) separation generation process. (b) Typical continuous voltage output of Ag/FEP/p-Si-based DD under the input frequency of 50 Hz. (c) Zoom-in voltage output of Ag/FEP/p-Si-based DD, showing a consistent resonance characteristic. (d) Typical continuous voltage output of Ag/FEP/n-Si-based DD under the input frequency of 50 Hz. The direction of the electricity generation is same with Ag/FEP/p-Si. (e) Zoom-in voltage output of Ag/FEP/n-Si-based DD, which also shows a consistent resonance characteristic. (f) Typical continuous voltage output of Ag/PTFE/p-Si-based DD under the input frequency of 50 Hz where PTFE is prepared without corona polarization. The generation phenomenon remains the same. (g) Zoom-in voltage output of Ag/PTFE/n-Si-based DD.

To explore the mechanical-electrical coupling process inside DD based on insulators, the response process from the perspective of time is systematically illustrated here; detailed results are shown in Figure 2. According to the physical model of DD, Fermi level/work function difference is vital to the generation direction of the device. In the previous report, the work function of PVDF/FEP/PTFE is calculated and tested to be ≈5.65/5.80/5.80 eV [24–26]; therefore, the DD based on these insulators and n/p silicon should produce electricity in the same direction as they possess work function ≈4-5 eV, which is shown in Figures 2(a) and 2(b). There are two parts of the generation process in the macroperspective: (1) contact and (2) separation, and the direction of the output voltage is different in two parts. In the first part, the hot holes (HHs) are excited and rebounded at the interface, and HCs are excited and rebounded in the second part. This whole process may be attributed to the mechanically excited HCs and strong interfacial electric field. To maintain the high energy level after conducting through thick bulk, the transport may have similar characters like hopping process; we propose interfacial rebound of hot carriers combined with the hopping-like physical process through the defects of polymer contribute to the electricity output inside insulator-based DD. Recently, Liu et al. report the possible mechanism of vertical/horizontal structure DD based on TiO_2_ and Al_2_O_3_, which are also well-known insulator materials. Interestingly, the TiO_2_-based device shows higher electricity output than Al_2_O_3_-based device of the same interfacial dielectric thickness (tens of nm) [27], which indicates that oxygen vacancy defect plays an important role in the carrier transport inside insulators. These results are also strong evidence for our proposed HCs rebound-hopping-like through insulator theory above. Intuitively speaking, rebounded HCs at the interface may ‘jump' through insulators to output electricity, thus subverting the stereotyped thinking of the insulators. Figures 2(b)–2(e) confirm the conjecture as both DDs show positive-negative signal within the same process of contact-separation. Figures 2(b) and 2(d) show the continuous stable output of the DD based on FEP/p-Si and FEP/n-Si, the voltage output is quite similar, and the zoom-in pictures of the output are shown in Figures 2(c) and 2(d). It can be observed that in general, the basic physical process is consistent with DD based on merely semiconductors; the work function difference can determine the direction of electricity output. In addition, this result further expands and optimizes the physical structure of DD; apart from diffusion and drift process, excitation and bulk transport is also one of the important processes after introducing insulators (dielectric materials) into the DD system. For ruling out the possibility of tribo/piezo related mechanism, DD based on p-Si/polytetrafluoroethylene (PTFE) film without corona polarization treatment is used here to determine the role of the fixed charges inside insulators, which are shown in Figures 2(f) and 2(g). To our knowledge, corona polarization is considered as one of the essential steps for manufacturing power generation devices based on insulators in the previous reports. Compared to FEP-based devices, the phenomenon remains the same with the same direction and similar voltage amplitude, proving the electricity is different from tribo/piezo-related mechanism as there are few fixed charges inside PTFE film here; in contrast, a larger amount of them in the FEP film was used here. Thus, unlike the piezo devices, the voltage difference induced by minor fixed charge movement inside bulk after applying external pressure is supposed to be the main generation mechanism; we propose the fixed charge inside insulator is not vital under DD structure; on the other hand, the mechanically induced HC excitation-transport process through the bulk may dominate the generation mechanism. However, the metal electrodes show better adhesion ability to insulators with corona polarization, so FEP is used more here for better stability. Uniquely, the device shows good relations with mechanical input signal in the frequency perspective. Here, the input frequency is settled as 50 Hz; the output also shows the same frequency with response time (from 10% of the peak output to 90%) of ~1 ms, leaving a wide silent region of >15 ms. This result indicates the DD based on insulators is a more promising candidate for mechanical sensing compared to similar devices.

**Figure 3:**
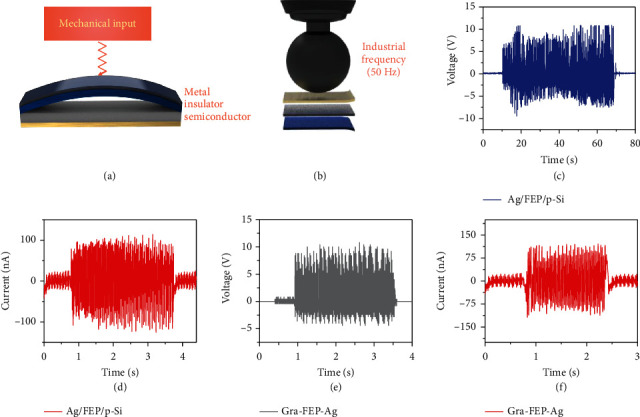
Daily application of the vertical DD. (a) Basic structure of practically vertical DD in the microperspective; a small gap is existed between insulator and semiconductor in the static mode. (b) Input of 50 Hz provided by daily used equipment. (c) Typical long-term steady output voltage under mechanical input of 50 Hz. (d) Typical long-term steady output current under mechanical input of 50 Hz for a single device. (e) Typical steady output voltage of a flexible DD based on Ag/FEP/graphene film. (f) Typical steady output current of a flexible DD based on Ag/FEP/graphene film.

For combining the device into self-powered electronic system, the essential frequency response characteristics must be systematically illustrated. Herein, input frequencies of various application scenarios are tested here. From Figure 2, we can observe that under the input frequency of sub-100 Hz, the response time is around 1 ms. As consequence, all those input mechanical signals can be effectively converted into electrical signal as shown in Figure 3. Figure 3(a) presents the microperspective of the device, where there is a small gap at the interface between insulator and semiconductor in the static/original state. After the mechanical input signal occurs, contact-separation movement can be achieved, which is equal to the collection of mechanical waves at the interface. The schematic diagram of the application structure is presented in Figure 3(b), where a daily mechanical source of 50 Hz is used here as the input, and the detailed output data are shown in Figures 3(c) and 3(d). Within a wide time region of more than 1 minute, the output shows a relatively stable characteristic in accordance to the input. Limited by the input condition, there are some fluctuations in the long-term input power density, leading to the indeterminate errors of the output. Figure 3(d) shows the output of the short-circuit current of a single device, where the working area is a magnitude smaller than Figure 1 due to the hard spherical contact. To enrich the application scenarios, flexible graphene film is used here to demonstrate the portable possibility, which may be helpful to promote the area such as real-time health detection, motion analysis, flexible electronic skin, and smart self-powered sensor system. Recently, some reports in the DD area illustrate the enhancement of output voltage, which is contributed to the unique carrier multiplication effect of graphene; however, single/few layers of graphene are needed to maintain the high energy level of HC [19]. Here, the flexible graphene film (~1000 layers) can also transport the highly energetic rebounded HC without significant loss, and a stable voltage output around ~10 V is reached for a single graphene film/FEP-based DD as shown in Figure 3(e). Figure 3(f) shows the current output for this single device, and the amplitude of the current under the same working area is near to the Si/FEP-based one, proving the limited amount but high energy HCs in the insulators.

**Figure 4:**
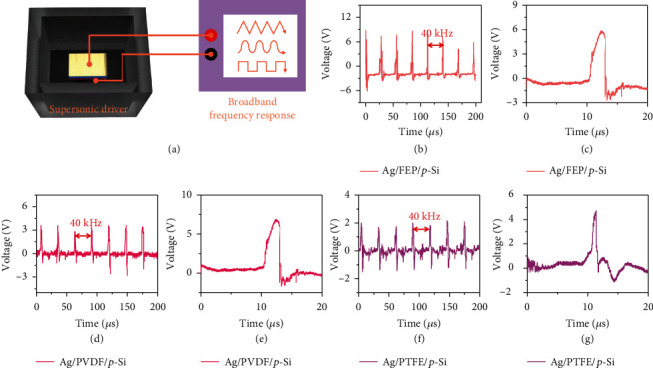
High-frequency mechanical signal collection ability of the DD based on insulator/semiconductor. (a) DD based on insulator/semiconductor inside an ultrasonic pool. (b) DD connected to oscilloscope through the top electrode and back electrode, which means the semiconductor is connected to input port (red) of the oscilloscope while insulator is connected to the output (black). (b) Typical voltage output of Ag/FEP/p-Si-based DD under the mechanical input frequency of 40 kHz. (c) Typical single voltage output of Ag/FEP/p-Si-based DD to verify the response time. (d) Typical voltage output of Ag/PVDF/p-Si-based DD under the mechanical input frequency of 40 kHz. (e) Typical single voltage output of Ag/PVDF/p-Si-based DD to verify the response time. (f) Typical voltage output of Ag/PTFE/p-Si-based DD under the mechanical input frequency of 40 kHz. (g) Typical single voltage output of Ag/PTFE/p-Si-based DD to verify the response time. The pressure here is tested to be 0.19 ± 0.01 N.

However, the macromechanical input fails to fully utilize the fast and wide frequency response potential. We assume this is attributed to the mechanical input mode as the human touch is a rather slow circle in the micro-perspective, which makes it more like a triangle signal input. As a result, we may have the question: what kind of response will DD based on insulators show under the input frequency above 1 kHz, if the output differs from the low frequency output, what response will high frequency input show? Will the generation of DD become direct current or show a highly consistent broadband resonance character? Therefore, the performance of supersonic (40 kHz) DD is systematically illustrated here. It is worth noting that the output maintains the alternative current output, and it shows an ultrafast response time of ~1 *μ*s. As shown in Figure 4, a commonly used supersonic driver is used here as the input, and the FEP/Si-based DD is placed inside the pool-alike environment to collect the micromechanical signal. The same with the experiments above, the top electrode from the insulator is connected to the input port (red) of oscilloscope, and the back electrode of the Si is connected to the output port (black) as shown in Figure 4(a); the detailed output is shown as below. Figure 4(b) is the output of the FEP/p-Si-based DD, a same frequency resonance is detected, and the zoom-in picture of a single pulse is shown in Figure 4(c). Here, as quite intriguing response time of ~1 *μ*s is illustrated, thus, there is a silent region of more than 20 *μ*s to ensure the resonance accuracy of 40 kHz input (25 *μ*s between two positive peaks). More interestingly, no significant decrease is detected between the daily macroscopic input frequencies and the microscopic input modes, indicating the promising future for this type of DD in the area of mechanical signal collection/analysis, which opens the avenue for combining macro mechanical signal to the modern electronic system. Figures 4(d)–4(g) show the output characteristics of PVDF/pSi-based and PTFE/pSi-based DD, which is consistent with the phenomenon above. From the experiments above, we assume that the DD based on insulators can respond to higher input frequency up to GHz, and the response time can be as short as the order of 1 ns.

## 3. Conclusion

In summary, an insulator/semiconductor-based DD was demonstrated as a promising candidate for combining macroscopic broadband mechanical signal into the modern electronic system. The excitation, rebound, and transport process of the HCs inside insulator-based DD is systematically discussed and illustrated here. Due to the unique structure of this type of DD, mechanically excited HCs at the interface between insulator/semiconductor can be efficiently rebounded and transport to output broadband resonance electrical signal, which opens up a new way to fully utilize the HCs inside DD. A maximum voltage response of 50 V and a stable resonance characteristic from 0-40 kHz is shown; besides, an ultrafast response time of 10^−6^ s (~1 *μ*s) is tested under the input frequency of 40 kHz, and the possible of the fastest response time can be predicted to ~1 ns. This work not only breaks the traditional mindset of the difference between insulator/conductor but also provides the evidence for the enriched application scenarios of DD. This device is a promising candidate for the self-powered smart sensor network, such as real-time health detection, motion analysis, flexible electronic skin, and underwater communication system.

## 4. Materials and Methods

### 4.1. Device Fabrication

Single side polished N-type and P-type doped silicon substrates were used, which were dipped into 10 wt% HF for 5 min to remove the native silicon oxide layer. The resistivity of P-type and N-type silicon was 0.5 *Ω* cm. The Ti/Au (5 nm/50 nm) electrode of the N-type or P-type silicon was grown using the magnetron sputtering method on the unpolished back side, which formed an ohmic contact after annealing in inert gas at a temperature of 400°C. The graphene membrane was fabricated with the tape casting method, in which the few-layer graphene powder was dispersed in water uniformly by the ultrasonic method, before heating and drying in a PET film. The ohmic electrode of the graphene membrane was fabricated with silver epoxy and drying with hot plate at a temperature of 110°C. Then, a plain conductor was connected to the silver electrode before being sealed using polydimethylsiloxane (PDMS) to avoid short circuiting. The insulators used here are bought from the Dupont Co., America (FEP&PVDF with corona polarization), and Tongshi Co., China (PTFE without corona polarization), and the electrodes are formed in the same way as a graphene film.

### 4.2. Characterization Analysis and Measurement

The image of the graphene membrane was recorded using a high definition camera. SEM images of the graphene membrane were recorded using a HITACHI S-4800 system. Keithley 2400 and Agilent B1500A systems were used to record the current–voltage (I–V) data. A Keithley 6514 system electrometer and DMM6500 multimeter were used to record the real time voltage and current responses of the vertical graphene/silicon DDG, which were controlled by a LabView-based data acquisition system with a sampling rate of 10 000 s^−1^. The high-frequency voltage output is tested by the GA1202EML oscilloscope with the sampling rate of 1GSa/s and bandwidth of 200 MHz.

## Data Availability

The data used to support the findings of this study are available from the corresponding author upon request.

## Supplementary Materials

Supplementary Materials Figure S1: (a) typical continuous voltage output of Cu/FEP/p-Si-based DD under the input frequency of 50 Hz. (b) Typical continuous voltage output of Au/FEP/p-Si-based DD under the input frequency of 50 Hz. (c) Typical continuous voltage output of Ag/FEP/Cu-based DD under the input frequency of 50 Hz. Figure S2: high-frequency mechanical signal collection ability of the DD based on insulator/semiconductor. (a) Typical voltage output of Au/FEP/p-Si-based DD under the mechanical input frequency of 40 kHz. (b) Typical single voltage output of Au/FEP/p-Si-based DD to verify the response time. Figure S3: typical current output of a single DD with the input of normal human contact. (a) DD based on Si/FEP/Ag film. (b) DD based on Ag/FEP/Graphene film. Figure S4: proposed energy band diagram inside DDG based on insulators/semiconductors heterostructure, while both PVDF and PTFE have a larger work function than n/p-Si. Figure S5: (a) voltage output of PVDF/p-Si-based DD and (b) voltage output of PVDF/n-Si-based DD. PVDF and n/p-Si-based DD show the same tendency with FEP and PTFE ones in the main text. Figure S6: the DD based on PVDF/pSi also has a higher output voltage under the extremely cold environment of 77 K, which may be attributed to the less scatter and higher mobility inside the bulk. Figure S7: the DD based on PVC (commonly used black insulation tape)/pSi also has an output under the framework of DD. Figure S8: molecular formula of insulators used in this work. (a) Fluorinated ethylene propylene, (b) polyvinylidene fluoride, and (c) polytetrafluoroethylene.
