# Supplementary material for: Broadband Insulator-Based Dynamic Diode with Ultrafast Hot Carriers Process
Source: Research (Wash D C). 2022 Sep 13;2022:9878352. doi: 10.34133/2022/9878352 (PMC9513832; doi:10.34133/2022/9878352)
Supplement: Supplementary Materials — Figure S1: (a) typical continuous voltage output of Cu/FEP/p-Si-based DD under the input frequency of 50 Hz. (b) Typical continuous voltage output of Au/FEP/p-Si-based DD under the input frequency of 50 Hz. (c) Typical continuous voltage output of Ag/FEP/Cu-based DD under the input frequency of 50 Hz. Figure S2: high-frequency mechanical signal collection ability of the DD based on insulator/semiconductor. (a) Typical voltage output of Au/FEP/p-Si-based DD under the mechanical input frequency of 40 kHz. (b) Typical single voltage output of Au/FEP/p-Si-based DD to verify the response time. Figure S3: typical current output of a single DD with the input of normal human contact. (a) DD based on Si/FEP/Ag film. (b) DD based on Ag/FEP/Graphene film. Figure S4: proposed energy band diagram inside DDG based on insulators/semiconductors heterostructure, while both PVDF and PTFE have a larger work function than n/p-Si. Figure S5: (a) voltage output of PVDF/p-Si-based DD and (b) voltage output of PVDF/n-Si-based DD. PVDF and n/p-Si-based DD show the same tendency with FEP and PTFE ones in the main text. Figure S6: the DD based on PVDF/pSi also has a higher output voltage under the extremely cold environment of 77 K, which may be attributed to the less scatter and higher mobility inside the bulk. Figure S7: the DD based on PVC (commonly used black insulation tape)/pSi also has an output under the framework of DD. Figure S8: molecular formula of insulators used in this work. (a) Fluorinated ethylene propylene, (b) polyvinylidene fluoride, and (c) polytetrafluoroethylene. [file 9878352.f1.docx]

**Supporting Information**

**Broadband Insulator-based Dynamic Diode with Ultrafast Hot Carriers Process**

Runjiang Shen^1^, Yanghua Lu^1^, Xutao Yu^1^, Qi Ge^2^, Huiming Zhong^3^, Shisheng Lin^1,2,4,5,*^

1 College of Information Science and Electronic Engineering, Zhejiang University, Hangzhou, 310027, China

2 Chongqing 2D Material Institute, Chongqing, 410020, China

3 Department of Emergency, The Second Affiliated Hospital, Zhejiang University School of Medicine, Zhejiang University, Hangzhou, 310009, China

4 State Key Laboratory of Modern Optical Instrumentation, Zhejiang University, Hangzhou, 310027, China

5 Hangzhou Gelanfeng Technology Co. Ltd, Hangzhou, 310051, China

*Corresponding author: shishenglin@zju.edu.cn


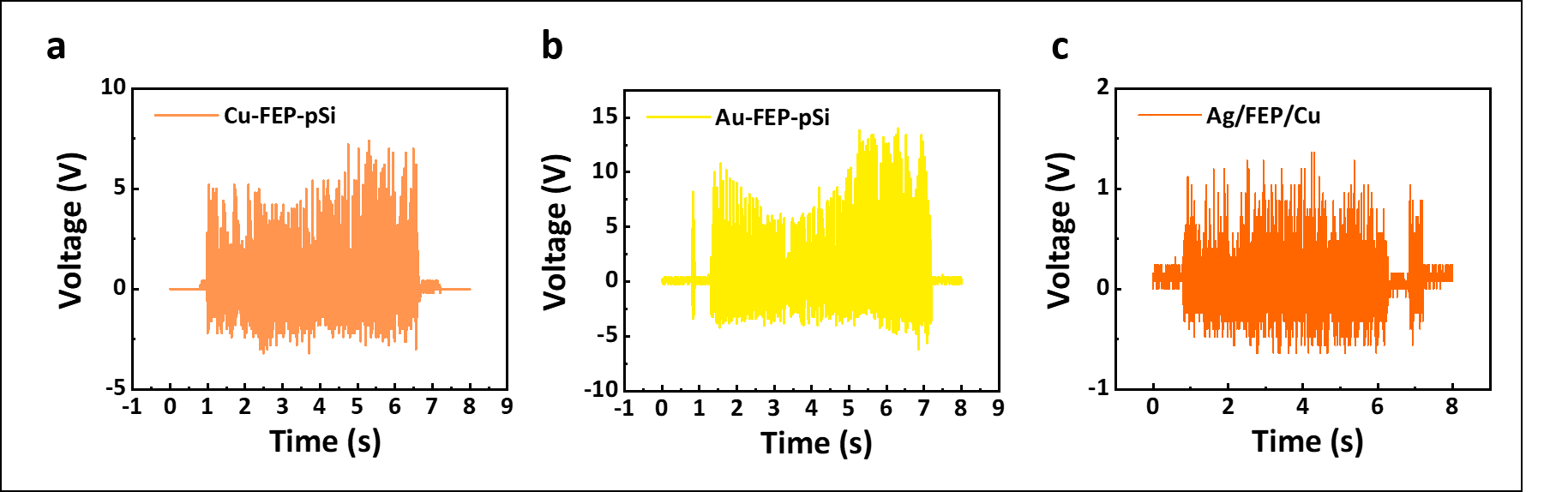


Figure S1. a) Typical continuous voltage output of Cu/FEP/p-Si based DD under the input frequency of 50Hz. b) Typical continuous voltage output of Au/FEP/p-Si based DD under the input frequency of 50Hz. c) Typical continuous voltage output of Ag/FEP/Cu based DD under the input frequency of 50Hz.


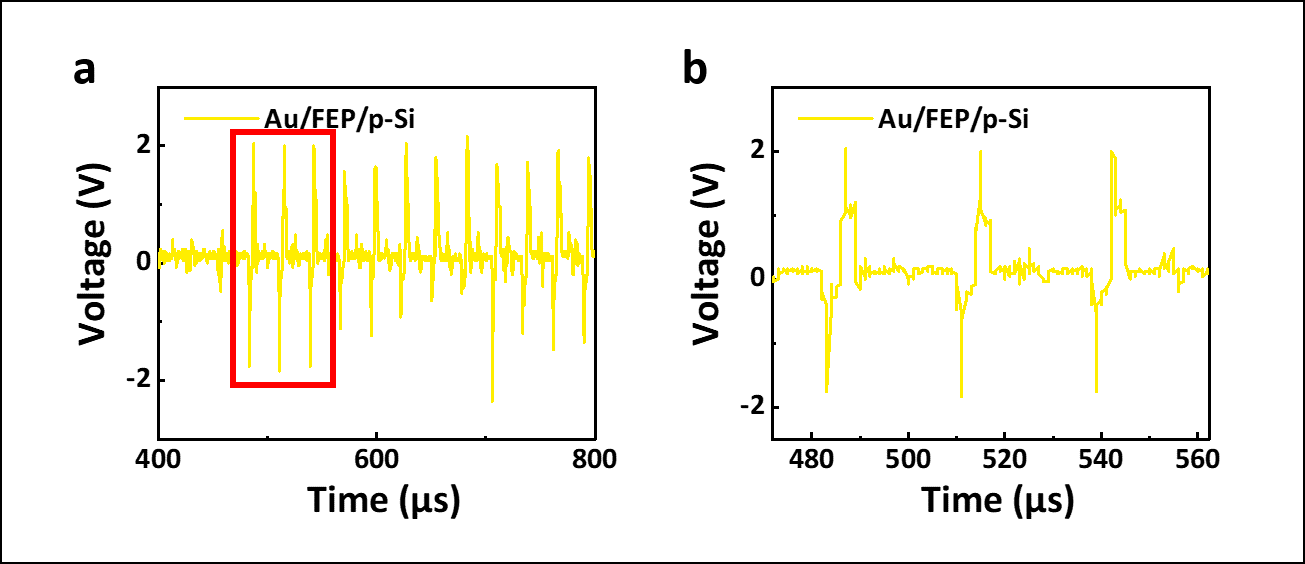


Figure S2. High frequency-mechanical signal collection ability of the DD based on insulator/semiconductor. a) Typical voltage output of Au/FEP/p-Si based DD under the mechanical input frequency of 40kHz. b) Typical single voltage output of Au/FEP/p-Si based DD to verify the response time.


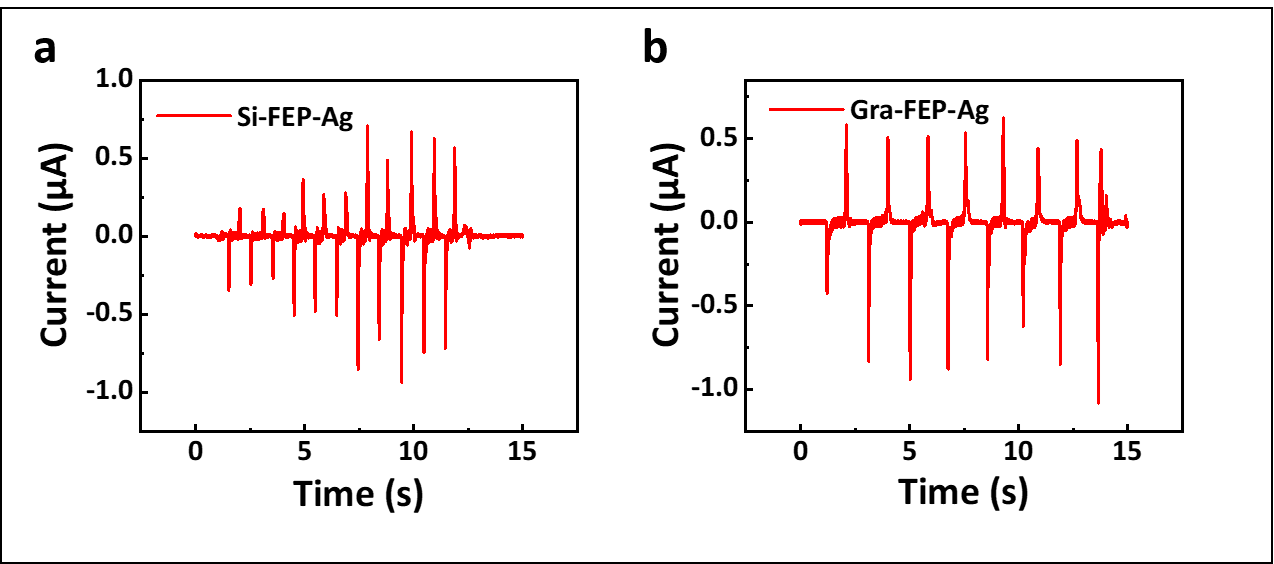


Figure S3. Typical current output of a single DD with the input of normal human contact a) DD based on Si/FEP/Ag film. b) DD based on Ag/FEP/Graphene film.


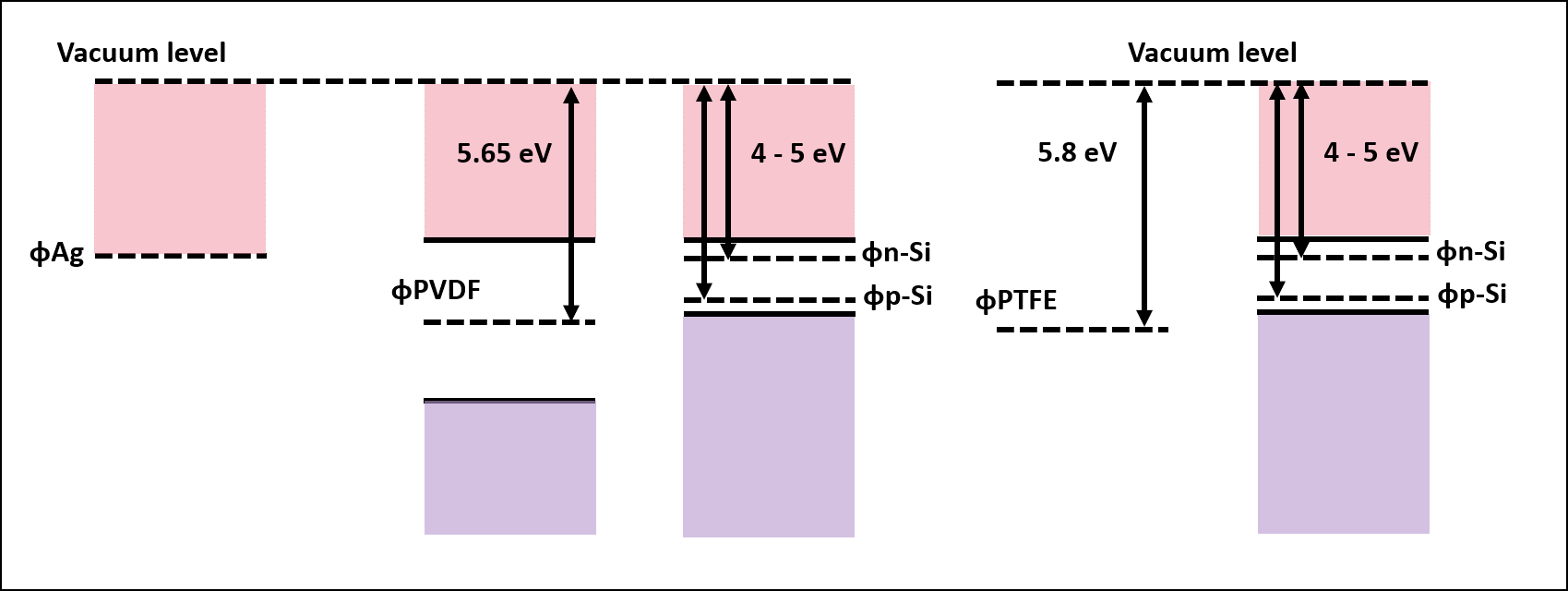


Figure S4. Proposed energy band diagram inside DDG based on insulators/semiconductors heterostructure, while both PVDF and PTFE have a larger work function than n/p-Si.


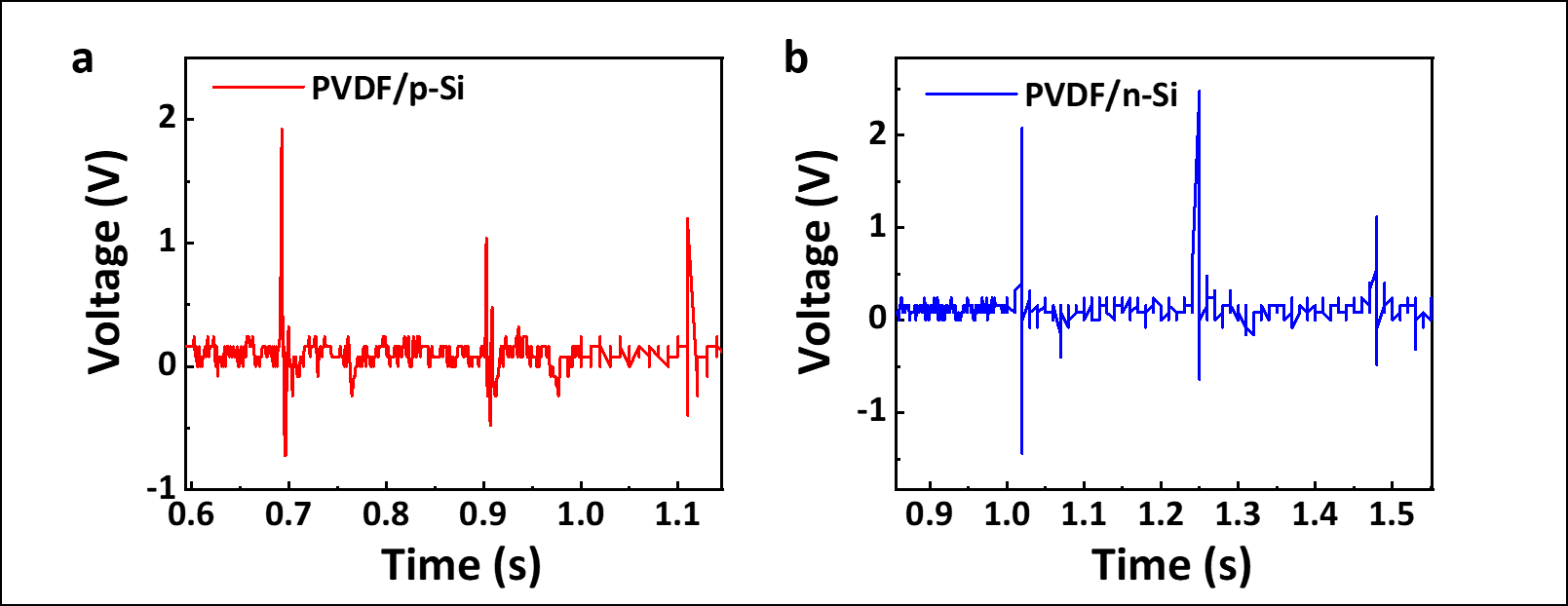


Figure S5. a) voltage output of PVDF/p-Si based DD, b) voltage output of PVDF/n-Si based DD. PVDF and n/p-Si based DD shows the same tendency with FEP and PTFE ones in the main text.


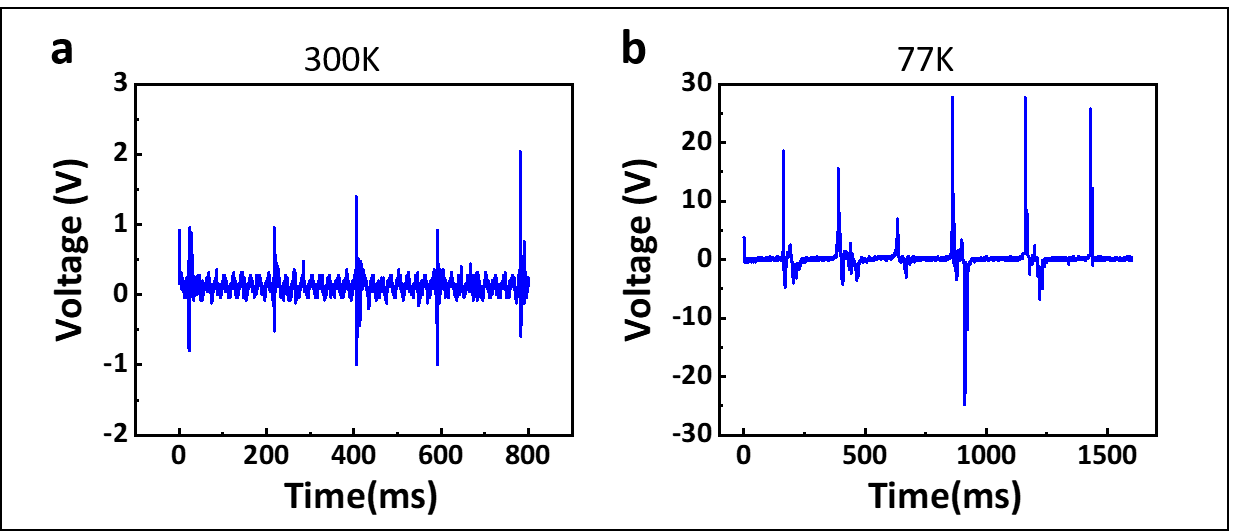


Figure S6. The DD based on PVDF/pSi also has a higher output voltage under the extremely cold environment of 77K, which may be attributed to the less scatter and higher mobility inside the bulk.


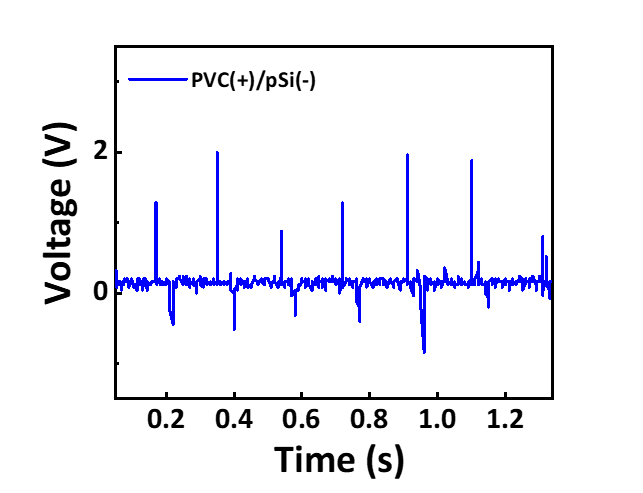


Figure S7. The DD based on PVC (commonly used black insulation tape)/pSi also has a output under the framework of DD.


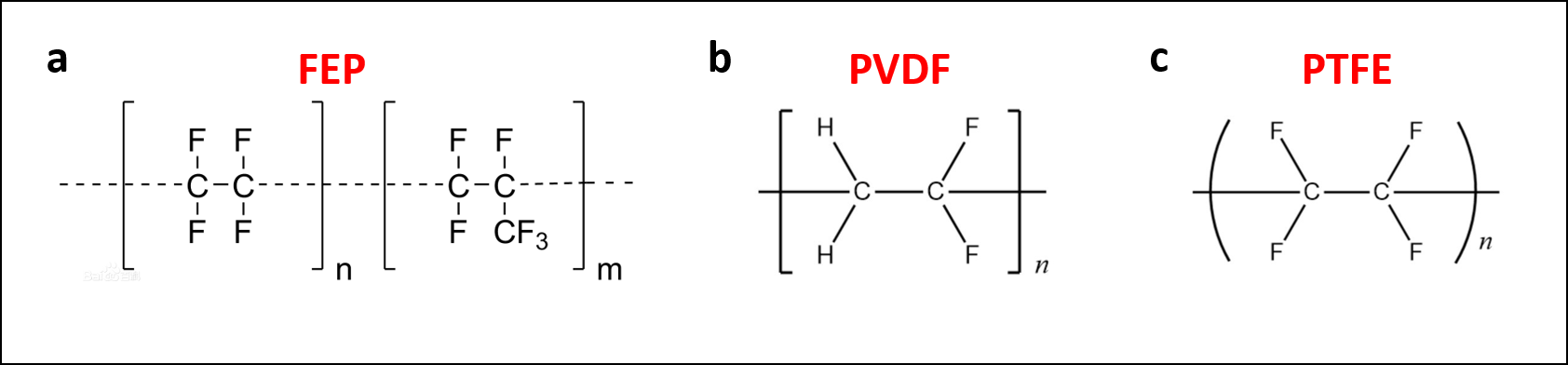


Figure S8. molecular formula of insulators used in this work. a) Fluorinated ethylene propylene, b) Polyvinylidene fluoride c) Poly tetra fluoroethylene
